# Differences in preferences for rural job postings between nursing students and practicing nurses: evidence from a discrete choice experiment in Lao People’s Democratic Republic

**DOI:** 10.1186/1478-4491-11-22

**Published:** 2013-05-24

**Authors:** Peter C Rockers, Wanda Jaskiewicz, Margaret E Kruk, Outavong Phathammavong, Phouthone Vangkonevilay, Chanthakhath Paphassarang, Inpong Thong Phachanh, Laura Wurts, Kate Tulenko

**Affiliations:** 1 Department of Global Health and Population, Harvard School of Public Health, 677 Huntington Ave, Boston, MA 02115, USA; 2 CapacityPlus, IntraHealth International Inc., Washington, DC, USA; 3 Department of Health Policy and Management, Mailman School of Public Health, Columbia University, New York, NY, USA; 4 World Health Organization, Vientiane, Lao People’s Democratic Republic; 5 Lao People’s Democratic Republic Ministry of Health, Vientiane, Lao People’s Democratic Republic

**Keywords:** Nursing, Human resources for health, Attraction, Retention, Discrete choice experiment, Laos People’s Democratic Republic

## Abstract

**Background:**

A discrete choice experiment was conducted to investigate preferences for job characteristics among nursing students and practicing nurses to determine how these groups vary in their respective preferences and to understand whether differing policies may be appropriate for each group.

**Methods:**

Participating students and workers were administered a discrete choice experiment that elicited preferences for attributes of potential job postings. Job attributes included salary, duration of service until promotion to permanent staff, duration of service until qualified for further study and scholarship, housing provision, transportation provision, and performance-based financial rewards. Mixed logit models were fit to the data to estimate stated preferences and willingness to pay for attributes. Finally, an interaction model was fit to formally investigate differences in preferences between nursing students and practicing nurses.

**Results:**

Data were collected from 256 nursing students and 249 practicing nurses. For both groups, choice of job posting was strongly influenced by salary and direct promotion to permanent staff. As compared to nursing students, practicing nurses had significantly lower preference for housing allowance and housing provision as well as lower preference for provision of transportation for work and personal use.

**Conclusions:**

In the Lao People’s Democratic Republic, nursing students and practicing nurses demonstrated important differences in their respective preferences for rural job posting attributes. This finding suggests that it may be important to differentiate between recruitment and retention policies when addressing human resources for health challenges in developing countries, such as Laos.

## Background

Lao People’s Democratic Republic (commonly referred to as Laos) is a lower-middle-income country located in Southeast Asia. Population health indicators in Laos have steadily improved over the past 15 years. Indeed, life expectancy increased from 51 years to 65 years between 1995 and 2008 and during that same time, infant mortality was more than halved from 102 to 42 deaths per 1,000 live births [1]. However, inequities in access to health care services between urban and rural populations threaten to undermine these achievements. The Laos Ministry of Health (MOH) is struggling to provide adequate access to primary health care services for rural populations that comprise more than three-quarters of the country’s total population. The MOH has identified a shortage of health workers at primary and secondary health facilities in rural areas as a priority area for health system reform efforts [2].

According to recent estimates, there are fewer than 5,000 nurses in Laos serving a population of more than six million, or less than one nurse per 1,000 population [1]. Further, as in many countries, a disproportionate number of nurses in Laos live and work in urban centers, leaving rural populations with limited access to skilled clinicians [3]. A recent study of the nurse workforce situation in Laos found that staffing shortages in the country do not stem from emigration [4]. The base salary for nurses in Laos in 2012 was 630,000 Lao Kip per month (around US$78.50). As of 2012, there were limited programs in place to incentivize nurses to live and work in rural areas. For example, there were no bonuses provided to nurses for working in rural areas, though such bonuses were under consideration by the MOH [5]. Further, at that time there were no conditional scholarship programs in the country that required nurses to fulfill a commitment to work in rural areas after receiving financial support for training.

The World Health Organization (WHO), the World Bank, USAID’s Capacity*Plus* project, and other partners have recently drafted a road map to guide governments in developing countries, such as Laos, in selecting and employing analytic tools to inform policy-making to effectively address human resources for health (HRH) challenges [6]. The draft road map identifies three important aspects of the problem of health worker staffing in underserved areas as critical for HRH policy: 1) understanding the health needs of the population and how the current health workforce is addressing or not addressing those needs; 2) understanding the health labor market; and 3) understanding the needs and expectations of health workers. Only after a thorough analysis of each of these aspects of the problem at the local-level can effective policy be formulated to address HRH challenges.

The discrete choice experiment (DCE) is one tool that policymakers can use to understand the needs and expectations of health workers. DCE is an analytic method that allows for the quantification of survey respondents’ preferences for various attributes of a good or service. DCEs that investigate health workers’ preferences for job posting attributes, including salary levels, health facility quality and future opportunities for advanced education and career promotion, have become increasingly common in the literature [7-13]. To support the method’s use in policy formulation, the World Bank, along with WHO and Capacity*Plus*, has developed a practitioner’s guide for conducting DCEs for HRH policy [14].

WHO has argued that governments should pursue both recruitment and retention policies to address health sector human resource constraints [15]. Recruitment policies primarily target students in the final year of health worker training programs, while retention programs target currently practicing health workers. Making decisions on investments in recruitment and retention strategies requires an understanding of the health labor market, for example, the available supply of graduating students in health worker training programs and the rates of turnover among current health sector employees. Determining the content of the incentive packages for recruitment and retention can be assisted by data from DCEs.

At present, there is little evidence to suggest how recruitment and retention policies for HRH should differ, if at all. Recently, several DCE studies have been published that focus alternatively on students in health worker training programs [3,4,8,9] or practicing health workers [5-7]. One DCE study, conducted by the World Bank in Vietnam and published in 2010, has compared preferences for job posting attributes among practicing physicians and medical students in their final year of training [16]. Further, a recent study in India employed qualitative methods to investigate differences in preferences for rural job postings among students and practicing health workers in doctor and nurse cadres [17]. However, further evidence is needed before firm conclusions can be drawn on this issue.

As an initial step toward identifying policies to address human resource constraints at rural health facilities, the Laos MOH solicited the assistance of WHO and Capacity*Plus* in determining preferences for rural job postings among students and health workers in the country. A DCE was conducted to investigate preferences for job characteristics among nursing students and practicing nurses. In this article we present a direct comparison of preferences for job postings among nursing students and practicing nurses in Laos. We aim to determine how students and health workers vary in their respective preferences to understand whether differing recruitment and retention policies may be appropriate.

## Methods

### Sampling

Three of 17 provinces in Laos were selected for inclusion in the study. One province each was selected from the northern (Luang Prabang), central (Savannakhet), and southern (Champasak) regions of the country. These particular provinces were selected for two reasons: 1) they were predominantly rural; and 2) they each included training program sites for health worker cadres of interest to the study. Within each selected province, nursing students and practicing nurses were recruited for the study according to specific methodologies.

#### Nursing students

The Laos government administers public nurse training programs at provincial colleges throughout the country. Each of the three provinces included in the study had nurse training programs at their respective provincial colleges. All students in their third and final year of the nurse training program in the three sampled provinces were invited to participate in this study.

#### Practicing nurses

Nurses practicing in Laos work at various levels of the health system, including the provincial hospital, district hospitals, and local health centers. Within each sampled province, all nurses at the provincial hospital were recruited for inclusion in the study. Further, four districts were selected from each province, and all nurses at the district hospitals and health centers within these districts were recruited for inclusion in the study. Provinces in Laos are comprised of five to ten districts.

### Instrument and survey fielding

Nursing students and practicing nurses completed identical DCE surveys that elicited preferences for attributes of nursing job postings in Laos. Selection of DCE attributes and levels was informed by three activities: 1) a review of the published literature on strategies to attract and retain health workers, 2) discussions with members of the Laos MOH, and 3) a focus group discussion with nursing students in Laos. For additional information on DCE instrument design see the Technical Appendix (Additional file 1). Six attributes were deemed sufficiently important to warrant inclusion in the final DCE instrument: salary, duration of service until promotion to permanent staff, duration of service until qualified for further study and scholarship, housing provision, transportation provision, and performance-based financial rewards (Table 1). DCE scenario alternatives were paired using an experimental design to optimize D-efficiency using Sawtooth Software’s Choice-Based Conjoint package (Sawtooth Software Inc. 2007). Five versions of the survey were generated and used in the study.

**Table 1:** DCE attributes and levels for nursing students and practicing nurses in Laos

Attribute 1	Salary^a^
Level 1	‘No additional salary’
Level 2	‘30% additional salary’
Level 3	‘40% additional salary’
Level 4	‘50% additional salary’
Attribute 2	Promotion to permanent staff
Level 1	‘Promoted to permanent staff after two years in rural facility’
Level 2	‘Promoted to permanent staff after one year in rural facility’
Level 3	‘Directly promoted to permanent staff upon posting in rural facility’
Attribute 3	Housing
Level 1	‘No housing provision’
Level 2	‘Housing allowance provided’
Level 3	‘Dormitory/housing provided’
Attribute 4	Duration of service before further study
Level 1	‘Qualify for further study and financial support after three years in rural facility’
Level 2	‘Qualify for further study and financial support after two years in rural facility’
Level 3	‘Qualify for further study and financial support after one year in rural facility’
Attribute 5	Transportation
Level 1	‘No transport provided’
Level 2	‘Transport provided for official activity/routine work’
Level 3	‘Transport provided for official and personal use’
Attribute 6	Performance-based financial award
Level 1	‘No bonus’
Level 2	‘Bonus for high performing nurse’

^a^Base salary for nurses in Laos at the time of survey administration: 630,000 LAK per month (1 USD = 8,025 LAK).

All participants were randomized to receive one of the five versions of the DCE instrument. For each version, respondents were presented with 12 choice tasks. Choice tasks were comprised of two job scenarios, each with a unique combination of levels for the six job attributes described above. For each choice task, respondents were asked, ‘Which of these two job postings do you prefer?’ The job postings described in the DCE scenarios were not labeled as being in rural or urban settings. Rather, as part of an introductory script presented prior to survey administration, respondents were told to consider all DCE job scenarios to be located in rural areas. No additional information on the definition of ‘rural’ was provided, so that respondents were free to interpret the term in their own way. There is some evidence that suggests that labels distract respondents from job attributes and thus may diminish the reliability of estimates of attribute preferences [18]. We were interested in investigating preferences for attributes of job postings within a rural setting. For additional information on DCE experimental design, see the Technical Appendix (Additional file 1). A survey questionnaire administered along with the DCE included questions about demographics, education experience, and prior work experience. Prior to the start of data collection, the DCE instrument was piloted with a small group of students to make certain that the presentation was conceptually clear.

Paper-based surveys were administered to student respondents in groups ranging from 50 to more than 150 respondents in classrooms, and to practicing health worker respondents in groups ranging from 10 to 20 at health facilities of employment during work hours. Respondents completed the survey questionnaire and DCE survey at their own pace. On average, respondents took approximately 20 to 30 minutes to complete the survey. At the completion of each survey group, the administrative team, comprised of members of the MOH, WHO, and Capacity*Plus*, entered the collected responses into project computers and aggregated the data into a central protected database.

The survey was fielded to inform health system reforms in Laos. All survey respondents provided written consent prior to participation. The Lao People’s Democratic Republic MoH subsequently shared data with the authors for analysis and publication. Clearance to analyze the de-identified administrative data and publish results was obtained by the institutional review board at Harvard University.

### Statistical analysis

Descriptive statistics were calculated for demographic and work experience variables. A separate main effects mixed logit model was fit to DCE data collected from each of the respondent groups - nursing students and practicing nurses - to estimate preferences for job attributes. In both models, all attribute variables were specified as having a random component except for salary, which was specified as fixed. Further, all attribute variables were coded as dummy variables except for salary, which was specified as continuous in both models. Mixed logit is not the only method that can be used to analyze DCE data, and there are ongoing debates in the literature regarding which modeling techniques are most efficient and appropriate [19]. Recent research suggests that traditional OLS modeling methods may be an efficient means to estimate individual-level choice preferences [20]. Mixed logit methods are frequently used for DCE modeling within the healthcare field [21,22]. However, recently generalized multinomial logit (GMNL) methods have been adopted in other fields that use the DCE method, including transportation economics [23]. The GMNL method separates heterogeneity in respondent preferences from heterogeneity in the scale of estimates produced by mixed logit models [24,25]. For comparison with estimates from mixed logit models, we also estimated the main results with GMNL models.

Willingness to pay (WTP) estimates were calculated by dividing attribute coefficients by the salary coefficient for both models. WTP confidence intervals were estimated using the delta method [26]. Finally, a formal statistical analysis of the differences in preferences for job posting attributes between nursing students and practicing nurses was conducted by fitting a mixed logit interaction model. The interaction model was fit to a DCE dataset that combined students’ and practicing nurses’ responses, and included a dummy variable that indicated that the respondent was a practicing nurse interacted with each of the DCE attributes. Interaction terms were specified as being fixed. A test for lexicographic preferences was conducted to identify respondents potentially behaving irrationally. For additional information on the statistical model specifications and the results of validity tests, see the Technical Appendix (Additional file 1). All mixed logit models were fit using Stata’s mixlogit command (StataCorp 2007), and were specified with 500 Halton draws.

## Results

Of 408 nursing students eligible for inclusion in the study, data were collected from 256 (62.7%). Of 383 practicing nurses eligible for inclusion in the study, data were collected from 249 (65.0%). Non-responses were due to eligible participants not reporting to survey locations. No data were collected from non-respondents.

Nursing students and practicing nurses were predominantly female (Table 2). The mean age of nursing students was 21.9 years (SD 2.7) and the mean age of practicing nurses was 39.2 years (SD 9.6). Most practicing nurses were married and had children while most nursing students were neither married nor had children. A majority of both nursing students (70.7%) and practicing nurses (65.1%) had lived in a rural area for at least one year at a previous time in their lives. Finally, as expected, practicing nurses had substantially more work experience than did nursing students. The median practicing nurse had worked 19 years at the time of the study while the median nursing students had not worked at all.

**Table 2:** **Descriptive statistics for students in the final year of nursing training programs and practicing nurses in Laos**, **2011**

		**Nursing students (N = 256)**		**Practicing nurses (N = 249)**	
		**n**	**(%)**	**n**	**(%)**
Demographics					
female		182	(71.1)	206	(82.7)
age, mean (SD)		21.9	(2.7)	39.2	(9.6)
currently married		11	(4.3)	211	(84.7)
has children		5	(2.0)	194	(77.9)
lived in rural area at least one year		181	(70.7)	162	(65.1)
Work experience					
years of work experience, median (IQR)		0	(0, 0)	19	(7, 24)
Facility level of current employment					
provincial hospital		-	-	81	(32.5)
district hospital		-	-	125	(50.2)
health center		-	-	43	(17.3)

Table 3 provides the output of two mixed logit models fit to DCE data collected from nursing students and practicing nurses in Laos. Mean utility coefficients (β) reflect relative preference weights where larger values indicate more preferred attributes. Mean utility coefficients from the nursing student model cannot be meaningfully compared to coefficients from the practicing nurse model because the utilities are on different scales. Mean utility coefficients are the basis for estimations of WTP, which can be compared across nursing students and practicing nurses (Table 4). Magnitudes of WTP estimates should be interpreted as willingness to give up salary in exchange for another attribute. Nursing students were most willing to give up salary in exchange for direct promotion to permanent staff, housing provision, and transportation provision for work and personal use. Practicing nurses had similar high willingness to give up salary in exchange for direct promotion to permanent staff. However, practicing nurses did not demonstrate a strong preference for housing provision nor for transportation provision for work and personal use. We present estimates of respondent preferences from GMNL models in Additional file 2. We find no substantive differences when comparing estimates from these models to estimates from mixed logit models, though the standard deviations, a measure of preference heterogeneity in the respondent population, produced by the GMNL models are consistently smaller.

**Table 3:** **Comparison of results from mixed logit models of DCE data from nursing students and practicing nurses in Laos**, **2011**

**Attribute**	**Nursing students**		**Practicing nurses**	
	**β**	**(SE)**	**β**	**(SE)**
duration of service until promoted to permanent staff (reference: two years)				
one year				
mean	0.585	(0.071)^c^	0.339	(0.064)^c^
SD	0.035	(0.199)	0.142	(0.229)
directly upon hiring				
mean	0.746	(0.098)^c^	0.692	(0.087)^c^
SD	1.029	(0.105)^c^	0.827	(0.097)^c^
duration of service until qualified for further study and scholarship (reference: three years)				
two years				
mean	0.231	(0.067)^c^	0.321	(0.064)^c^
SD	0.019	(0.146)	0.008	(0.138)
one year				
mean	0.459	(0.078)^c^	0.286	(0.067)^c^
SD	0.595	(0.105)^c^	0.339	(0.129)^c^
housing (reference: none)				
housing allowance				
mean	0.566	(0.076)^c^	0.324	(0.066)^c^
SD	0.449	(0.117)^c^	0.263	(0.151)^a^
housing provided				
mean	0.714	(0.077)^c^	0.395	(0.066)^c^
SD	0.427	(0.116)^c^	0.243	(0.162)
transportation (reference: none)				
provided for work purposes only				
mean	0.544	(0.071)^c^	0.439	(0.067)^c^
SD	0.155	(0.213)	0.244	(0.156)
provided for work and personal use				
mean	0.775	(0.079)^c^	0.432	(0.068)^c^
SD	0.417	(0.126)^c^	0.318	(0.133)^b^
performance-based financial award (reference: none)				
mean	0.403	(0.065)^c^	0.396	(0.063)^c^
SD	0.689	(0.079)^c^	0.670	(0.077)^c^
salary (% change above base^d^)				
mean	0.019	(0.002)^c^	0.019	(0.002)^c^
alternative-specific constant				
mean	0.096	(0.052)^a^	0.041	(0.046)
Model diagnostics				
number of respondents	256		249	
number of observations	6,112		5,952	
log likelihood	−1,814.3		−1,825.3	
likelihood ratio χ^2^	< 0.001		< 0.001	

^a^*P* < 0.10, ^b^*P* < 0.05, ^c^*P* < 0.01.

^d^Base salary for nurses in Lao PDR at the time of survey administration: 630,000 LAK per month (1 USD = 8,025 LAK).

**Table 4:** **Willingness to pay for job attributes among nursing students and practicing nurses in Laos**, **2011**

**Attribute**	**Nursing students**	**Practicing nurses**
	**% base salary**^**a**^	**% base salary**
duration of service until promoted to permanent staff (reference: two years)		
one year	30.174	18.078
	(22.730, 37.619)	(11.302, 24.854)
directly upon hiring	38.503	36.958
	(27.515, 49.492)	(26.995, 46.921)
duration of service until qualified for further study and scholarship (reference: three years)		
two years	11.899	17.140
	(5.033, 18.764)	(10.273, 24.008)
one year	23.698	15.275
	(15.181, 32.215)	(8.081, 22.468)
housing (reference: none)		
housing allowance	29.224	17.320
	(20.328, 38.121)	(9.792, 24.847)
housing provided	36.841	21.097
	(27.592, 46.091)	(13.554, 28.641)
transportation (reference: none)		
provided for work purposes only	28.071	23.451
	(20.366, 35.776)	(16.087, 30.814)
provided for work and personal use	39.992	23.071
	(30.803, 49.182)	(15.490, 30.652)
performance-based financial award	20.767	21.113
	(13.569, 27.966)	(13.984, 28.243)
number of respondents	256	249
number of observations	6,112	5,942

^a^Base salary for nurses in Laos at the time of survey administration: 630,000 LAK per month (1 USD = 8,025 LAK). Numbers in parentheses are 95% confidence intervals.

Table 5 presents a formal analysis of differences in preferences for job attributes between nursing students and practicing nurses. The results of the interaction model confirm that, compared to nursing students, practicing nurses had a significantly lower preference for housing allowance and provision as well as transportation provision for personal use. In addition, practicing nurses had a significantly lower preference for promotion to permanent staff after one year of work as compared to two years than did nursing students. As with the main effect model estimates, interaction estimates from GMNL models were not substantively different from mixed logit estimates, though the standard errors produced by the GMNL models were consistently larger (see Additional file 2).

**Table 5:** **Interaction model demonstrating differences in preferences for job posting attributes between nursing students and practicing nurses in Laos**, **2011**

**Interaction**	**β**	**(SE)**
Practicing nurse X		
duration of service until promoted to permanent staff (reference: two years)		
one year	−0.193	(0.092)^b^
directly upon hiring	−0.007	(0.125)
duration of service until qualified for further study and scholarship (reference: three years)		
two years	0.087	(0.090)
one year	−0.165	(0.100)^a^
housing (reference: none)		
housing allowance	−0.191	(0.096)^b^
housing provided	−0.254	(0.095)^c^
transportation (reference: none)		
provided for work purposes only	−0.075	(0.094)
provided for work and personal use	−0.285	(0.099)^c^
performance-based financial award (reference: none)	0.029	(0.088)
salary (% change above base)	0.001	(0.002)
Model diagnostics		
number of respondents	505	
number of observations	12,064	
log likelihood	−3,644.3	
likelihood ratio χ^2^	< 0.001	

^a^*P* < 0.10, ^b^*P* < 0.05, ^c^*P* < 0.01.

## Discussion

We investigated similarities and differences in preferences for rural job postings among nursing students and practicing nurses in Laos. This information can help the Laos MOH better understand which strategies to pursue to address HRH challenges in rural areas in the country. Nursing students and practicing nurses had similar preferences for several job posting attributes. Both groups were willing to give up more than 35% of their monthly salary in exchange for being promoted to permanent staff directly upon hiring. Similarly, both groups of respondents were willing to give up around 21 to 23% of their monthly salary in exchange for the institution of a performance-based financial reward for high performing nurses.

Despite these similarities, there were important differences in preferences for job posting attributes between nursing students and practicing nurses in Laos. Practicing nurses placed significantly lower value on housing provision as compared to nursing students. In a recent DCE that looked at practicing nurses’ preferences for job attributes in Kenya, South Africa and Thailand, Blaauw *et al*. found that respondents’ preference for housing attributes were consistently lower than were preference for other attributes such as training opportunities and salary increases [4]. Further, as compared to nursing students, practicing nurses placed significantly lower value on transportation provision for work and personal use.

There are several plausible mechanisms that could explain why we observe differences in preferences for job posting attributes. First, practicing nurses in this study were on average 39 years old and the median nurse had been in the workforce 19 years, and thus likely had substantial experience in obtaining housing and arranging transportation. On the other hand, lack of housing and transportation might be much more daunting to a nursing student contemplating a rural job, resulting in higher utilities for these incentives among students. In addition to differences in job experience, younger nursing students and older practicing nurses are in different stages in their lives, such that what they consider to be most important may be very different. Indeed, very few of the nursing students had families, while a large majority of practicing nurses were married and had children. Finally, not all nursing students choose to pursue a career in the nursing profession upon completion of their studies. Similarly, some nurses leave the nursing profession early in their career. Therefore, the practicing nurses included in this study may constitute a self-selected sample with unmeasured characteristics that influence both the probability of remaining a practicing nurse and preferences for job posting attributes.

With the data we have collected for this study, we unfortunately have limited ability to differentiate the potential effects of these proposed mechanisms. We find that, among practicing nurses, age was not significantly associated with preferences for job attributes (see Additional file 3). This may validate the self-selection explanation. There is, however, evidence from the labor economics literature that work experiences do indeed influence preferences for future job opportunities as well as actual selection of jobs [27,28]. The literature suggests that one reason preferences differ is because experienced workers have more information upon which to base decisions [29]. However, related research on health workers in low-income settings is limited. Future investigations should aim to understand how age, experience, and states of life might influence preferences and job decisions.

The findings we present in this study suggest that it may be important to differentiate recruitment and retention policies when addressing HRH challenges in developing countries, such as Laos. There is support for this finding in the literature. Rao *et al*. found, in a qualitative study conducted in India, that students in training to become doctors and nurses had different preferences for rural job postings as compared to practicing doctors and nurses [13]. Further, in an analysis comparing preferences for job postings among medical students and practicing physicians in Vietnam, Vujicic *et al*. found both similarities and differences in preferences for job attributes between student and professional groups [12]. The authors concluded that governments should tailor HRH attraction and retention strategies to doctors at different stages of their careers. We do not, however, present data in this paper that may indicate whether recruitment or retention policies should be prioritized at this time by the government of Laos. In order to make a strong argument for specific policies, it would be necessary to collect and analyze information related to the health needs of the population and the health worker labor market in the country.

This analysis had important limitations. First, we did not collect the data necessary to fully delineate the explanatory power of two plausible mechanisms - work experience and self-selection - on observed differences in preferences for job attributes between nursing students and practicing nurses. Second, we did not collect information from non-respondents and thus were unable to assess whether selection bias may have resulted from differential participation. However, because surveys were administered to students during class hours and to practicing nurses during work hours, we may conclude that the primary reason for non-response was unavailability due to class and work responsibilities. Further, we would not expect such a selection mechanism to significantly bias the observed results. Third, the DCE presented to nursing students and practicing nurses in Laos was unlabeled. That is, DCE scenarios were not identified as being in urban or rural settings. As such, we can’t comment on the effect of rural job location on respondents’ job preferences. Fourth, the hypothetical nature of the DCE methods employed in this study may raise validity concerns. Respondents may not have fully understood the survey directions and may not have accurately stated their preferences. Similarly, respondents may have made choices based on what they felt interviewers wanted them to choose, biasing results. Perhaps most importantly, it is unlikely that the preference data that we present comprises the full range of factors that determine nurses’ job-related decision making in Laos. Recent research suggests that, among workers across various economic sectors, factors related to job satisfaction do not explain a substantial proportion of job turnover [30]. Future research should focus on validating DCE results from analyses of health worker data with information on revealed preferences, that is, observations of actual health worker job choice behavior. However, it is difficult to disaggregate job attributes to permit the analysis of their separate influence in real-life scenarios.

## Conclusions

The Laos MOH is seeking to address HRH challenges to meet the health needs of the country’s rural populations. The challenges faced in Laos are not only local, they are global. Developing and developed countries throughout the world are struggling with similar challenges. Further, while emigration is not currently a primary contributor to staffing shortage in Laos, it is a substantial concern in other countries, underscoring the cross-border nature of the issue. International solutions to the challenges of health worker staffing are required [31]. This study demonstrates the potential value that the DCE method can provide to HRH policymakers. In Laos, conducting a thorough analysis of the local labor market -including production rates and attrition - would help to determine whether recruitment or retention policies (or what mixture of the two) may be most effective in filling vacant nurse postings in underserved areas. The DCE information presented here may be used in conjunction with labor market data to inform the development of specific policy interventions.

## Abbreviations

DCE: Discrete choice experiment; HRH: Human resources for health; MOH: Ministry of Health; USAID: United States Agency for International Development; WHO: World Health Organization; WTP: Willingness to pay.

## Competing interests

The authors declare that they have no competing interests.

## Author’ contributions

PCR contributed to the design of the study and led the analysis and writing. WJ conceived the study and assisted with data collection and writing. MEK assisted with study design and writing. OP, PV, CP, and ITP assisted with study design, data collection and writing. LW assisted with the data collection and writing and KT assisted with study design, analysis and writing. All authors read and approved the final manuscript.

## Supplementary Material

Additional file 1 Technical Appendix. Click here for file

Additional file 2 Estimates from generalized multinomial logit (GMNL) models. Click here for file

Additional file 3 Effect of age on preferences among practicing nurses. Click here for file
