# Supplementary material for: Differences in preferences for rural job postings between nursing students and practicing nurses: evidence from a discrete choice experiment in Lao People’s Democratic Republic
Source: Hum Resour Health. 2013 May 24;11:22. doi: 10.1186/1478-4491-11-22 (PMC3671159; doi:10.1186/1478-4491-11-22)
Supplement: Additional file 1 — Technical Appendix. [file 1478-4491-11-22-S1.docx]

**Additional file 1.** Technical Appendix

*Instrument design and administration*

Identification of DCE attributes and levels: The selection of DCE scenario attributes and levels was informed by three activities: 1) a review of the published literature on strategies to attract and retain health workers, 2) discussions with members of the Lao People’s Democratic Republic (commonly referred to as Laos) Ministry of Health (MOH), and 3) a focus group discussion with practicing nurses in Laos.

Several articles describing DCEs related to human resources for health (HRH) have been published in the past few years. These articles include information about health workers’ preferences for rural job postings in developing countries throughout the world. Lagarde and Blaauw have recently published a review of this work [1]. These articles and a recent WHO report about financial and non-financial incentives for HRH attraction and retention were used to devise an initial list of potentially important job attributes for health workers in Laos [2]. Then, through meetings with key members of the Laos Ministry of Health, those attributes that were most relevant in the Laos context were identified.

Finally, a focus group discussion (FGD) was held with nursing students in Laos. All FGD participants gave written consent to participate. FGD participants were first asked general questions about their perceptions of health worker job postings in Laos. Next, participants discussed factors that they considered to be important to them when they thought about where they would most prefer to work. Participants were asked specific questions about job attributes that were previously identified based on the literature review and meetings with the Laos MOH, as described above. Participants were asked to rank attributes according to importance. For each attribute, participants were asked to identify levels that were realistic and appropriate in the local context.

Based on information collected during FGDs, six attributes were included in the final DCE instrument: salary, duration of service until promotion to permanent staff, duration of service until qualified for further study and scholarship, housing provision, transportation provision, and performance-based financial rewards.

Selection of scenarios: The number of possible job posting scenarios is calculated as a function of the number of attributes and levels. The DCE administered in this study had one attribute with four levels, four attributes with three levels and one attribute with two levels. This design generated 648 (4^1^ x 3^4^ x 2^1^) possible scenarios. The magnitude of possible scenarios is too many for any individual respondent to evaluate. As such, DCE scenario alternatives were paired using an experimental design to maximize level balance and orthogonality (i.e., to optimize D-efficiency), and to minimize overlap among attribute levels. This was done using Sawtooth Software’s Choice-Based Conjoint package (Sawtooth Software Inc. 2007). In describing the algorithm that the software uses to achieve an efficient design, Sawtooth describes each of the primary conditions [3]. With level balance, “each level of an attribute is shown approximately an equal number of times.” According to the orthogonality condition, “attribute levels are chosen independently of other attribute levels, so that each attribute level’s effect (utility) may be measured independently of all other effects.” Finally, with minimal overlap, “each attribute level is shown as few times possible in a single task. If an attribute’s number of levels is equal to the number of product concepts in a task, each level is shown exactly once.” Five versions of the survey were generated and used in the study. We present the two-way frequencies for each attribute level in the final surveys in Table A1.1. Further, we present estimates of the study design’s efficiency in Table A1.2. The estimates presented in the table indicate the *a priori* standard error for the attribute level based on the study design as a proportion of ideal standard error that maximizes statistical power.

**Table A1.1.** Two-way frequencies of attributes levels in the study design

| Attribute/level^1^ | 1/1 | 1/2 | 1/3 | 1/4 | 2/1 | 2/2 | 2/3 | 3/1 | 3/2 | 3/3 | 4/1 | 4/2 | 4/3 | 5/1 | 5/2 | 5/3 | 6/1 | 6/2 |
| --- | --- | --- | --- | --- | --- | --- | --- | --- | --- | --- | --- | --- | --- | --- | --- | --- | --- | --- |
| 1/1 | 30 | 0 | 0 | 0 | 10 | 9 | 11 | 11 | 10 | 9 | 10 | 9 | 11 | 10 | 10 | 10 | 16 | 14 |
| 1/2 | 0 | 30 | 0 | 0 | 10 | 10 | 10 | 10 | 10 | 10 | 10 | 10 | 10 | 10 | 10 | 10 | 14 | 16 |
| 1/3 | 0 | 0 | 30 | 0 | 10 | 10 | 10 | 10 | 9 | 11 | 10 | 10 | 10 | 10 | 10 | 10 | 15 | 15 |
| 1/4 | 0 | 0 | 0 | 30 | 10 | 11 | 9 | 9 | 11 | 10 | 10 | 11 | 9 | 10 | 10 | 10 | 15 | 15 |
| 2/1 | 10 | 10 | 10 | 10 | 40 | 0 | 0 | 14 | 12 | 14 | 13 | 13 | 14 | 13 | 14 | 13 | 19 | 21 |
| 2/2 | 9 | 10 | 10 | 11 | 0 | 40 | 0 | 14 | 14 | 12 | 14 | 14 | 12 | 14 | 13 | 13 | 20 | 20 |
| 2/3 | 11 | 10 | 10 | 9 | 0 | 0 | 40 | 12 | 14 | 14 | 13 | 13 | 14 | 13 | 13 | 14 | 21 | 19 |
| 3/1 | 11 | 10 | 10 | 9 | 14 | 14 | 12 | 40 | 0 | 0 | 12 | 13 | 15 | 13 | 14 | 13 | 20 | 20 |
| 3/2 | 10 | 10 | 9 | 11 | 12 | 14 | 14 | 0 | 40 | 0 | 15 | 14 | 11 | 13 | 14 | 13 | 20 | 20 |
| 3/3 | 9 | 10 | 11 | 10 | 14 | 12 | 14 | 0 | 0 | 40 | 13 | 13 | 14 | 14 | 12 | 14 | 20 | 20 |
| 4/1 | 10 | 10 | 10 | 10 | 13 | 14 | 13 | 12 | 15 | 13 | 40 | 0 | 0 | 13 | 13 | 14 | 10 | 21 |
| 4/2 | 9 | 10 | 10 | 11 | 13 | 14 | 13 | 13 | 14 | 13 | 0 | 40 | 0 | 14 | 13 | 13 | 20 | 20 |
| 4/3 | 11 | 10 | 10 | 9 | 14 | 12 | 14 | 15 | 11 | 14 | 0 | 0 | 40 | 13 | 14 | 13 | 21 | 19 |
| 5/1 | 10 | 10 | 10 | 10 | 13 | 14 | 13 | 13 | 13 | 14 | 13 | 14 | 13 | 40 | 0 | 0 | 20 | 20 |
| 5/2 | 10 | 10 | 10 | 10 | 14 | 13 | 13 | 14 | 14 | 12 | 13 | 13 | 14 | 0 | 40 | 0 | 20 | 20 |
| 5/3 | 10 | 10 | 10 | 10 | 13 | 13 | 14 | 13 | 13 | 14 | 14 | 13 | 13 | 0 | 0 | 40 | 20 | 20 |
| 6/1 | 16 | 14 | 15 | 15 | 19 | 20 | 21 | 20 | 20 | 20 | 19 | 20 | 21 | 20 | 20 | 20 | 60 | 0 |
| 6/2 | 14 | 16 | 15 | 15 | 21 | 20 | 19 | 20 | 20 | 20 | 21 | 20 | 19 | 20 | 20 | 20 | 0 | 60 |

^1^Attribute and level numbering correspond to the ordering presented in Table A1.2

**Table A1.2.** Estimates of study design efficiency

| Attribute and level | | *A priori* standard error | Efficiency |
| --- | --- | --- | --- |
| Salary | |  |  |
|  | “No additional salary” | Ref |  |
|  | “30% additional salary” | 0.305 | 1.075 |
|  | “40% additional salary” | 0.345 | 0.843 |
|  | “50% additional salary” | 0.329 | 0.923 |
| Promotion to permanent staff | |  |  |
|  | “Promoted to permanent staff after 2 years in rural facility” | Ref |  |
|  | “Promoted to permanent staff after 1 year in rural facility” | 0.261 | 0.977 |
|  | “Directly promoted to permanent staff upon posting in rural facility” | 0.260 | 0.984 |
| Housing | |  |  |
|  | “No housing provision” | Ref |  |
|  | “Housing allowance provided | 0.262 | 0.973 |
|  | “Dormitory/housing provided” | 0.263 | 0.963 |
| Duration of service before further study | |  |  |
|  | “Qualify for further study and financial support after 3 years in rural facility” | Ref |  |
|  | “Qualify for further study and financial support after 2 years in rural facility” | 0.262 | 0.975 |
|  | “Qualify for further study and financial support after 1 year in rural facility” | 0.262 | 0.969 |
| Transportation | |  |  |
|  | “No transport provided” | Ref |  |
|  | “Transport provided for official activity/routine work” | 0.263 | 0.962 |
|  | “Transport provided for official and personal use” | 0.263 | 0.967 |
| Performance-based financial award | |  |  |
|  | “No bonus” | Ref |  |
|  | “Bonus for high performing nurse” | 0.183 | 0.991 |

Fielding: Data collection activities were conducted during May 2011. Paper-based surveys were administered to student respondents in groups ranging from 50 to more than 150 respondents in classrooms, and to practicing health worker respondents in groups ranging from 10 to 20 at health facilities of employment during work hours. All participants were randomized to receive one of the five versions of the DCE instrument. Prior to beginning the DCE, all respondents were read a standard introductory script by study personnel. The purpose of the script was to acclimate respondents to the hypothetical nature of the DCE they were about to take. Respondents were asked to imagine making a real choice considering only the attributes described. Further, respondents were instructed that there were no right or wrong answers. Respondents then proceeded to complete the survey questionnaire and DCE at their own pace. Respondents were presented with 12 random tasks, each comprised of two job scenarios. Respondents were asked to select their preferred scenario. On average, respondents took approximately 20 to 30 minutes to complete the survey. All respondents provided consent prior to participating in the study.

*Statistical analysis*

We fit a main effects mixed logit model to the collected DCE data. All attribute variables were specified as having a random component except for salary, which was specified as fixed in all models. Specifying salary as fixed is preferred for calculation and interpretation of willingness to pay [4]. All attribute variables were coded as dummy variables except for salary, which was specified as continuous. An alternative-specific constant was included in all models. A test for dominant preferences was conducted to identify respondents potentially behaving irrationally. Output from mixed logit models includes two parameter estimates: mean utility and standard deviation. Mean utility coefficients reflect relative preference weights where larger values indicate more preferred attributes. Standard deviation estimates reflect heterogeneity in respondent preferences [5]. All mixed logit models were fit using Stata’s mixlogit command, and were specified with 500 Halton draws (StataCorp 2007).

*Test for lexicographic preferences*

The data were tested to identify respondents who always selected a job posting on the basis of one attribute irrespective of the levels of other attributes, i.e., lexicographic preferences. While lexicographic preferences imply non-compensatory behavior, they do not imply irrationality [6]. Further, evidence of lexicographic preferences may reflect weaknesses in study design rather than respondent decision making rules. In our data, we found that among both nursing students and practicing nurses there were no respondents with lexicographic preferences.

**Technical appendix references**

1. Lagarde M, Blaauw D: **A review of the application and contribution of discrete choice experiments to inform human resources policy interventions.** *Hum Resour Health* 2009, **7:**62.

2. World Health Organization: **Increasing access to health workers in remote and rural areas through improved retention.** Geneva: WHO; 2010.

3. Sawtooth Software Inc: **The CBC System for Choice-Based Conjoint Analysis, Version 8. Technical Paper Series. Sawtooth Software, Utah.** 2013.

4. Revelt D, Train K: **Mixed logit with repeated choices: households' choices of appliance efficiency levels.** *Review of Economics and Statistics* 1998, **80:**647-657.

5. Kjaer T, Gyrd-Hansen D: **Preference heterogeneity and choice of cardiac rehabilitation program: results from a discrete choice experiment.** *Health Policy* 2008, **85:**124-132.

6. Lancsar E, Louviere J: **Deleting 'irrational' responses from discrete choice experiments: a case of investigating or imposing preferences?***Health Economics* 2006, **15:**797-811.
