# Supplementary material for: Differences in preferences for rural job postings between nursing students and practicing nurses: evidence from a discrete choice experiment in Lao People’s Democratic Republic
Source: Hum Resour Health. 2013 May 24;11:22. doi: 10.1186/1478-4491-11-22 (PMC3671159; doi:10.1186/1478-4491-11-22)
Supplement: Additional file 2 — Estimates from generalized multinomial logit (GMNL) models. [file 1478-4491-11-22-S2.docx]

**Additional file 2.** Estimates from generalized multinomial logit (GMNL) models

**Table A2.1.** Comparison of preference estimates from GMNL models

| **Attribute** | | | | **Nursing**  **students** | | **Practicing**  **nurses** | | |
| --- | --- | --- | --- | --- | --- | --- | --- | --- |
|  |  | | | **β** | **(SE)** | **β** | | **(SE)** |
| Duration of service until promoted to permanent staff (ref: 2 years) | | | |  |  |  | |  |
|  | | 1 year | |  |  |  | |  |
|  | | | Mean | 0.649 | (0.093)*** | 0.335 | | (0.113)*** |
|  | | | SD | 0.042 | (0.123) | 0.134 | | (0.087) |
|  | | Directly upon hiring | |  |  |  | |  |
|  | | | Mean | 0.805 | (0.126)*** | 0.790 | | (0.178)*** |
|  | | | SD | 0.536 | (0.206)*** | 0.413 | | (0.207)** |
| Duration of service until qualified for further study and scholarship  (ref: 3 years) | | | |  |  |  | |  |
|  | | 2 years | |  |  |  | |  |
|  | | | Mean | 0.275 | (0.075)*** | 0.376 | | (0.098)*** |
|  | | | SD | 0.010 | (0.075) | 0.007 | | (0.058) |
|  | | 1 year | |  |  |  | |  |
|  | | | Mean | 0.540 | (0.095)*** | 0.410 | | (0.106)*** |
|  | | | SD | 0.280 | (0.125)** | 0.184 | | (0.104)* |
| Housing (ref: none) | | | |  |  |  | |  |
|  | | Housing allowance | |  |  |  | |  |
|  | | | Mean | 0.669 | (0.097)*** | 0.512 | | (0.123)*** |
|  | | | SD | 0.120 | (0.093) | 0.093 | | (0.098) |
|  | | Housing provided | |  |  |  | |  |
|  | | | Mean | 0.787 | (0.104)*** | 0.670 | | (0.151)*** |
|  | | | SD | 0.226 | (0.101)** | 0.128 | | (0.097) |
| Transportation (ref: none) | | | |  |  |  | |  |
|  | | Provided for work purposes only | |  |  |  | |  |
|  | | | Mean | 0.639 | (0.095)*** | 0.803 | | (0.177)*** |
|  | | | SD | 0.118 | (0.089) | 0.140 | | (0.084)* |
|  | | Provided for work and personal use | |  |  |  | |  |
|  | | | Mean | 0.973 | (0.120)*** | 0.858 | | (0.177)*** |
|  | | | SD | 0.101 | (0.139) | 0.007 | | (0.135) |
| Performance-based financial award (ref: none) | | | |  |  |  | |  |
|  | | | Mean | 0.408 | (0074)*** | 0.514 | | (0.115)*** |
|  | | | SD | 0.347 | (0.146)** | 0.337 | | (0.165)** |
| Salary (% change above base^1^) | | | |  |  |  | |  |
|  | | | Mean | 0.023 | (0.003)*** | 0.029 | | (0.006)*** |
|  |  | | |  | |  | | |
| Alternative-specific constant | | | | 0.110 | (0.059)* | 0.024 | (0.056) | |
|  |  | | |  | |  | | |
| **Model diagnostics** | | | |  | |  | | |
|  | Number of respondents | | | 256 | | 249 | | |
|  | Number of observations | | | 6,112 | | 5,952 | | |
|  | Log likelihood | | | -1,788.2 | | -1,777.9 | | |
|  | Likelihood ratio χ^2^ | | | < 0.001 | | < 0.001 | | |

*P<0.10, **P<0.05, ***P<0.01

^1^Base salary for nurses at the time of survey administration: 630,000 LAK per mo. (1 USD = 8,025 LAK)

**Table A2.2.** Willingness to pay using estimates from GMNL models

| **Attribute** | | | **Nursing**  **students** | **Practicing**  **nurses** |
| --- | --- | --- | --- | --- |
|  | | | **% base salary^1^**  **(95% CI)** | **% base salary**  **(95% CI)** |
| Duration of service until promoted to permanent staff (ref: 2 years) | | |  |  |
|  | | 1 year | 27.798  (21.435, 34.161) | 12.118  (6.469, 17.768) |
|  | | Directly upon hiring | 34.523  (25.128, 43.918) | 27.277  (20.509, 34.044) |
| Duration of service until qualified for further study and scholarship (ref: 3 years) | | |  |  |
|  | | 2 years | 11.775  (5.521, 18.029) | 12.777  (7.454, 18.100) |
|  | | 1 year | 23.157  (15.576, 30.738) | 13.691  (8.120, 19.262) |
| Housing (ref: none) | | |  |  |
|  | | Housing allowance | 28.676  (21.133, 36.219) | 17.459  (11.805, 23.113) |
|  | | Housing provided | 33.720  (25.840, 41.600) | 22.976  (16.936, 29.016) |
|  | Transportation (ref: none) | |  |  |
|  | | Provided for work purposes only | 27.373  (20.551, 34.194) | 27.794  (21.865, 33.723) |
|  | | Provided for work and personal use | 41.707  (34.113, 49.301) | 29.246  (23.438, 35.055) |
|  | Performance-based financial award | | 17.471  (11.760, 23.181) | 17.566  (12.434, 22.698) |
|  |  | |  |  |
| Number of respondents | | | 256 | 249 |
| Number of observations | | | 6,112 | 5,942 |

^1^Base salary for nurses in Laos at the time of survey administration: 630,000 LAK per month (1 USD = 8,025 LAK)**Table A2.3.** Interaction estimates from a GMNL model

| **Interaction** | | | **β** | **(SE)** |
| --- | --- | --- | --- | --- |
| **Practicing nurse X** | | |  |  |
|  | Duration of service until promoted to permanent staff (ref: 2 years) | |  |  |
|  | | 1 year | -0.310 | (0.127)** |
|  | | Directly upon hiring | -0.089 | (0.163) |
|  | Duration of service until qualified for further study and scholarship (ref: 3 years) | |  |  |
|  | | 2 years | 0.057 | (0.109) |
|  | | 1 year | -0.198 | (0.126) |
|  | Housing (ref: none) | |  |  |
|  | | Housing allowance | -0.173 | (0.116)^1^ |
|  | | Housing provided | -0.188 | (0.132)^2^ |
|  | Transportation (ref: none) | |  |  |
|  | | Provided for work purposes only | 0.106 | (0.139) |
|  | | Provided for work and personal use | -0.261 | (0.152)* |
|  | Performance-based financial award (ref: none) | | 0.056 | (0.101) |
|  | Salary (% change above base) | | 0.004 | (0.004) |
|  | | |  |  |
| **Model diagnostics** | | |  |  |
|  | Number of respondents | | 505 | |
|  | Number of observations | | 12,064 | |
|  | Log likelihood | | -3,573.2 | |
|  | Likelihood ratio χ^2^ | | < 0.001 | |

*P<0.10, **P<0.05, ***P<0.01

^1^P-value = 0.13

^2^P-value = 0.16
