# Supplementary material for: Differences in preferences for rural job postings between nursing students and practicing nurses: evidence from a discrete choice experiment in Lao People’s Democratic Republic
Source: Hum Resour Health. 2013 May 24;11:22. doi: 10.1186/1478-4491-11-22 (PMC3671159; doi:10.1186/1478-4491-11-22)
Supplement: Additional file 3 — Effect of age on preferences among practicing nurses. [file 1478-4491-11-22-S3.docx]

**Additional File 3.** Effect of age on preferences among practicing nurses

**Table A3.1.** Interaction model demonstrating differences in preferences for job posting attributes among practicing nurses by age in Laos, 2011

| **Interaction** | | | **β** | **(SE)** |
| --- | --- | --- | --- | --- |
| **Age of practicing nurse X** | | |  |  |
|  | Duration of service until promoted to permanent staff (ref: 2 years) | |  |  |
|  | | 1 year | -0.014 | (0.010) |
|  | | Directly upon hiring | -0.005 | (0.009) |
|  | Duration of service until qualified for further study and scholarship (ref: 3 years) | |  |  |
|  | | 2 years | -0.0004 | (0.007) |
|  | | 1 year | 0.004 | (0.007) |
|  | Housing (ref: none) | |  |  |
|  | | Housing allowance | -0.008 | (0.008) |
|  | | Housing provided | -0.004 | (0.008) |
|  | Transportation (ref: none) | |  |  |
|  | | Provided for work purposes only | -0.007 | (0.007) |
|  | | Provided for work and personal use | -0.004 | (0.007) |
|  | Performance-based financial award (ref: none) | | -0.001 | (0.006) |
|  | Salary (% change above base) | | -0.0002 | (0.0002) |
|  | | |  |  |
| **Model diagnostics** | | |  |  |
|  | Number of respondents | | 242 | |
|  | Number of observations | | 5,784 | |
|  | Log likelihood | | -1,768.2 | |
|  | Likelihood ratio χ^2^ | | < 0.001 | |

*P<0.10, **P<0.05, ***P<0.01
